# Investigation of a two‐step device implementing magnetophoresis and dielectrophoresis for separation of circulating tumor cells from blood cells

**DOI:** 10.1002/elsc.202000001

**Published:** 2020-04-27

**Authors:** Amir Shamloo, Alireza Yazdani, Fatemeh Saghafifar

**Affiliations:** ^1^ Department of Mechanical Engineering Sharif University of Technology Tehran Iran

**Keywords:** cell separation, circulating tumor cells, dielectrophoresis, magnetophoresis, microfluidic

## Abstract

Identifying tumor cells from a pool of other cells has always been an appealing topic for different purposes. The objective of this study is to discriminate circulating tumor cells (CTCs) from blood cells for diagnostic purposes in a novel microfluidic device using two active methods: magnetophoresis and dielectrophoresis. The most specific feature of this device is the differentiation of CTCs without labeling them in order to achieve a more reliable and less complicated method. This device was analyzed and evaluated using finite element method. Four cell lines are separated in this device containing red blood cells, platelets, white blood cells, and CTCs. Primarily, red blood cells and platelets, which constitute the largest part of a blood sample, are removed in the magnetophoresis section. Remaining cells enter the dielectrophoresis part and based on their inherent dielectric properties and diameters, final separation occurs. In each step, different parameters are examined to obtain the maximum purification. The results demonstrate the potential of different CTCs separation by changing the effective parameters in the designed device based on the inherent properties of the cells.

**Abbreviations**
CTC: circulating tumor cell
DEP: dielectrophoresis
MAP: magnetophoresis
MCFAV: maximum possible cell flow average velocity
PLT: platelet
RBC: red blood cell

## 1 INTRODUCTION

Cell separation and sorting has recently become a helpful method in biological and medical applications for three major purposes: diagnostics, therapeutics, and cell biology [1]. For such processes, different methods and devices are evaluated based on their accuracy, throughput, and time consumption. These advantages can be achieved using microfluidic‐based sorters.

Cell recognition methods in these sorters can be divided into three main categories: fluorescent label‐based, bead‐based (like magnetic‐activated), and label‐free cell sorting. Among these methods, label‐free cell sorting has some advantages. Label‐free sorting relies on the differences between physical properties of the particles such as their shape, size, and magnetic susceptibility [2]. These methods reduce the needed time and cost for cell sorting and also require minimum preparation [2, 3]. Label‐free cell sorting can also be used in both active and passive methods, while the others are only applicable in active methods [2].

In cell separation, methods including external fields in order to impose forces on particles, such as dielectrophoresis (DEP) and magnetophoresis (MAP), are called active methods and the others that do not include such external field, like inertial forces and filters, are called passive methods [2]. Passive methods can only differentiate the cells with different size, shape, compressibility, or density, whereas active methods can sort the cells with the same size or shape.

DEP for cell sorting was introduced by Pohl in 1966 [4]. In that study, it was defined how dielectrophoresis is different from electrophoresis and also DEP force was used to cause selective migration of living and dead yeast cells where living cells separated and remained viable. Separation using DEP can be used even in submicron particles such as viruses as shown by Hughes et al. [5]. In that study, some microelectrode arrays were used to separate submicron latex spheres. Here, the separation happens because of different DEP forces imposed on different particles as a result of change in their surface and dielectric properties.

Cell sorting using a single active method has been conducted in different studies. For example, Piacentini et al. designed a device that separates red blood cells (RBCs) from platelets (PLTs) using dielectrophoresis‐field‐flow‐fractionation, where they were able to obtain a purification of almost 99% for PLTs [6]. Another example could be the separation of viable and nonviable yeast cells using DEP [7]. The problem is that the DEP force without any enrichment stage could generate Joule heating in blood sample and it is also not sufficient enough to separate more than two types of the cells. MAP force could be used as a preenrichment stage of DEP separation in order to enhance the efficiency and purity of separation.

In the present study, a new approach for separating circulating tumor cells (CTCs) from other blood cells is introduced in a two‐step microfluidic device. In the first section, a geometry for MAP separation is developed using a permanent magnet that leads to the separation of RBCs and PLTs, which are the dominant blood cells in population, from the whole blood sample. Afterward, WBCs and CTCs are separated based on their inherent electric properties using dielectrophoresis‐field‐flow‐fractionation method. In this device, CTCs are separated with a label‐free method to let them be minimally manipulated. Active methods have been used in this study since it has been demonstrated that some CTCs and leukocytes have almost the same size and that could be problematic for the differentiation of CTCs from blood cells [8].

## 2 SEPARATION MECHANISM

**FIGURE 1:**
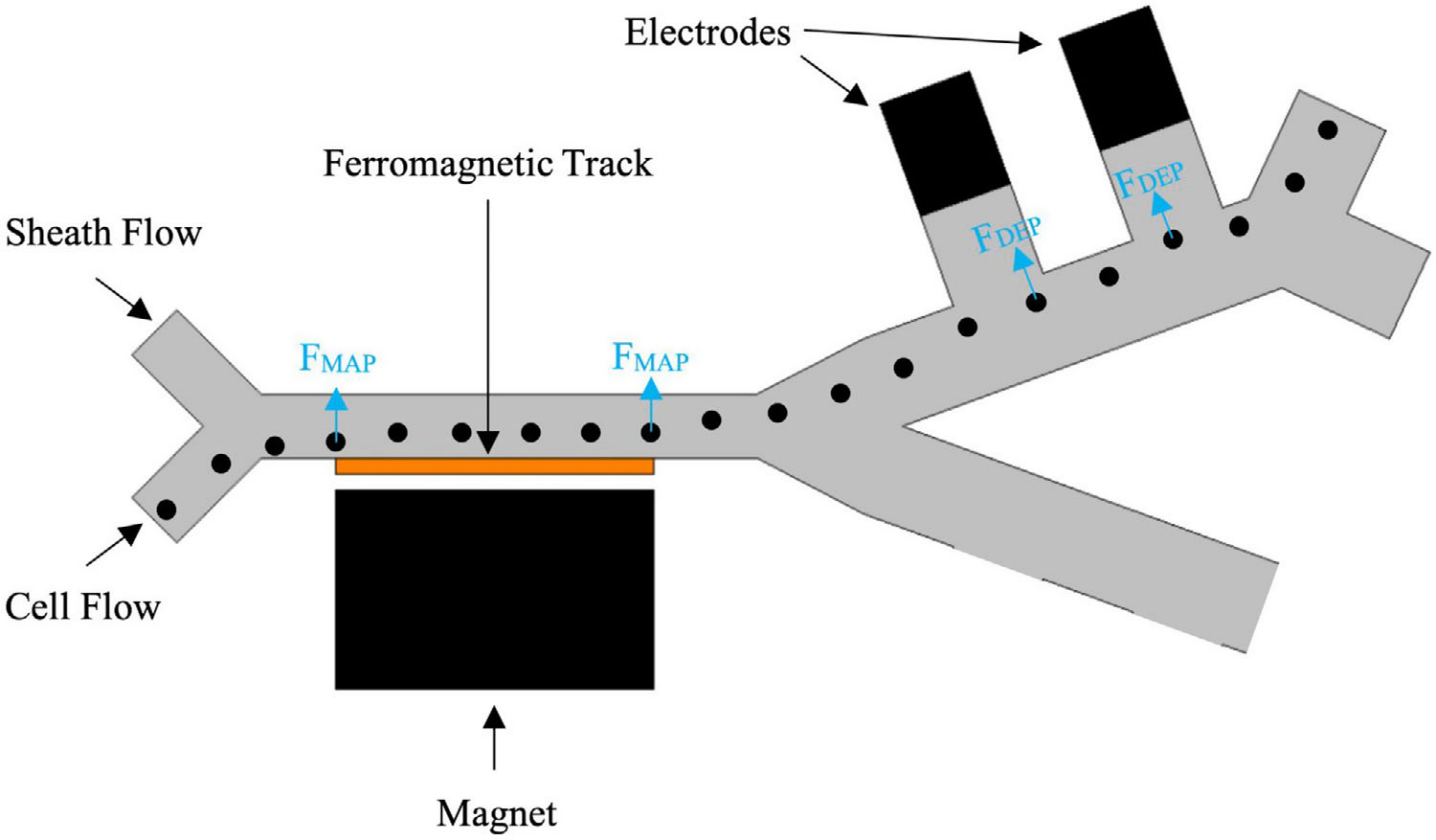
2D Schematic of the device

The proposed microfluidic device consists of two different sections. The first section uses magnetophoretic force in order to separate cells, while the next section utilizes dielectrophoretic force. In the proposed device, the bigger cells (WBCs and CTCs) were separated from the smaller ones (RBCs and PLTs) in the magnetophoretic section, and in the dielectrophoretic section, the separation of WBCs and CTCs from each other was performed. The schematic design of the device is depicted in Figure 1.

MAP has been proposed to show the behavior of magnetic particles when they are placed in a viscous medium and a nonuniform external magnetic field. There are three limiting cases in MAP, which can be found extensively in the work of Zborowski et al. [9]. Since most of the biological particles are weakly paramagnetic, they become linearly polarized magnetic dipole in an external magnetic field. In this case, magnetic volume susceptibility is independent of the effective parameters. The exerted force on such particles can be defined as [9]:

(1) $$F_{\mathrm{MAP}}=\left(\chi_{\mathrm{cell}}-\chi_{\mathrm{medium}}\right)V\nabla E\;,$$

where

(2) $$E=\frac{1}{2}BH\;.$$

Hence, the force on a paramagnetic spherical particle (with radius *r*) can be defined as:

(3) $$F_{\mathrm{MAP}}=\frac{4}{3}\pi r^{3}\left(\chi_{\mathrm{cell}}-\chi_{\mathrm{medium}}\right)H\nabla B\;,$$

where χ represents susceptibility, *H* represents magnetic field, and *B* is magnetic flux density. If the cell susceptibility is greater than the medium susceptibility, positive magnetophoretic force is imposed on the cell. Otherwise, a negative force is exerted on the cell called negative magnetophoretic force.

Dielectrophoretic force separates different cells based on their size and electric properties. In a harmonic electric field, dielectric particles become polarized, and subsequently, electric field inside and outside of the particle will have different values than the harmonic field. Gascoyne et al. [10] showed the relation between the fields inside and outside of the particle. Then they found the dielectrophoretic force by using Maxwell stress tensor. If a biological particle is floating in a medium and the sample is placed in a slightly nonuniform electric field, it suffices to use the first order of Taylor expansion. Finally, the force exerted on the particle (with radius *r*) is [10]:

(4) FDEP=2πr3εmReCM∇Erms2,

where $\varepsilon_{m}$ is the medium permittivity, CM is Clausius–Mossoti factor, and $E_{rms}\;$is the electric field. CM is defined by the following equation [10]:

(5) $$\mathrm{CM}=\frac{\varepsilon_{p}^{*}-\varepsilon_{m}^{*}}{\varepsilon_{p}^{*}+2\varepsilon_{m}^{*}}\;,$$

where $\varepsilon_{p}^{*}$ and $\varepsilon_{m}^{*}$ are complex permittivity of particle and medium, respectively. considering a medium with spherical particles inside, complex permittivity is defined by the following equation:

(6) $$\varepsilon^{*}=\varepsilon -\frac{\sigma }{\omega }i\;,$$

where ε is permittivity, σ is conductivity, and ω is the frequency of nonhomogeneous harmonic electric field.

Equation (5) indicates that when the complex permittivity of particles is greater than the medium, the force is positive, and it drags the particles in the direction of increasing electric field (pDEP). Otherwise, the particles are repelled from the electrodes (nDEP). This parameter is a function of frequency of the electric field, explaining the importance of frequency of applied voltage to achieve separation. There are several studies that use the frequency to manipulate the viable particles in travelling electric fields [6, 11].

**FIGURE 2:**
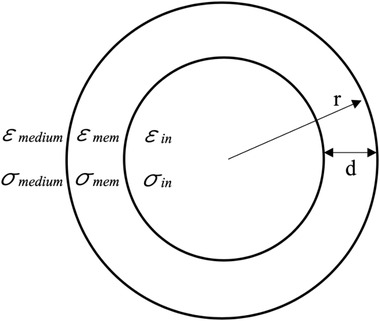
Single shell model

Our simulations are based on the single‐shell model, in which an equivalent permittivity is assumed for the particle in terms of complex permittivity of the core and the shell. This equivalent permittivity is defined as [12]:

(7) $$\varepsilon_{\mathrm{eff}}^{*}=\varepsilon_{\mathrm{mem}}^{*}\frac{{\left(\frac{r}{r-d}\right)}^{3}+2\left(\frac{\varepsilon_{\mathrm{in}}^{*}-\varepsilon_{\mathrm{mem}}^{*}}{\varepsilon_{\mathrm{in}}^{*}+2\varepsilon_{\mathrm{mem}}^{*}}\right)}{{\left(\frac{r}{r-d}\right)}^{3}-\left(\frac{\varepsilon_{\mathrm{in}}^{*}-\varepsilon_{\mathrm{mem}}^{*}}{\varepsilon_{\mathrm{in}}^{*}+2\varepsilon_{\mathrm{mem}}^{*}}\right)}\;,$$

where $\varepsilon_{eff}^{*}$ will be replaced by $\varepsilon_{p}^{*}$ for particle's permittivity. All of the parameters are defined in Figure 2.

Drag force is a hydrodynamic force that opposes the particle relative movement. Reynolds number in our simulations is small, thus we use Stokes’ drag equation:

(8) $$D=6\pi \mu r\left(U-V\right)\;,$$

where *μ* is medium viscosity, *U* is particle velocity, and *V* is the fluid velocity. Finally, after the computation of the force field within the domain, we use the following equation to obtain the particles trajectory:

(9) $$F_{\mathrm{Total}}=\frac{d\left(mv\right)}{dt}\;.$$

Finally, by solving the ordinary and partial differential equations of magnetic, electric and fluid flow parts, desired fields are obtained, and subsequently, we are able to compute the magnetophoretic, dielectrophoretic, and drag forces. These equations are not mentioned in this article to keep it short and brief.

## 3 NUMERICAL MODELING AND RESULTS

**FIGURE 3:**
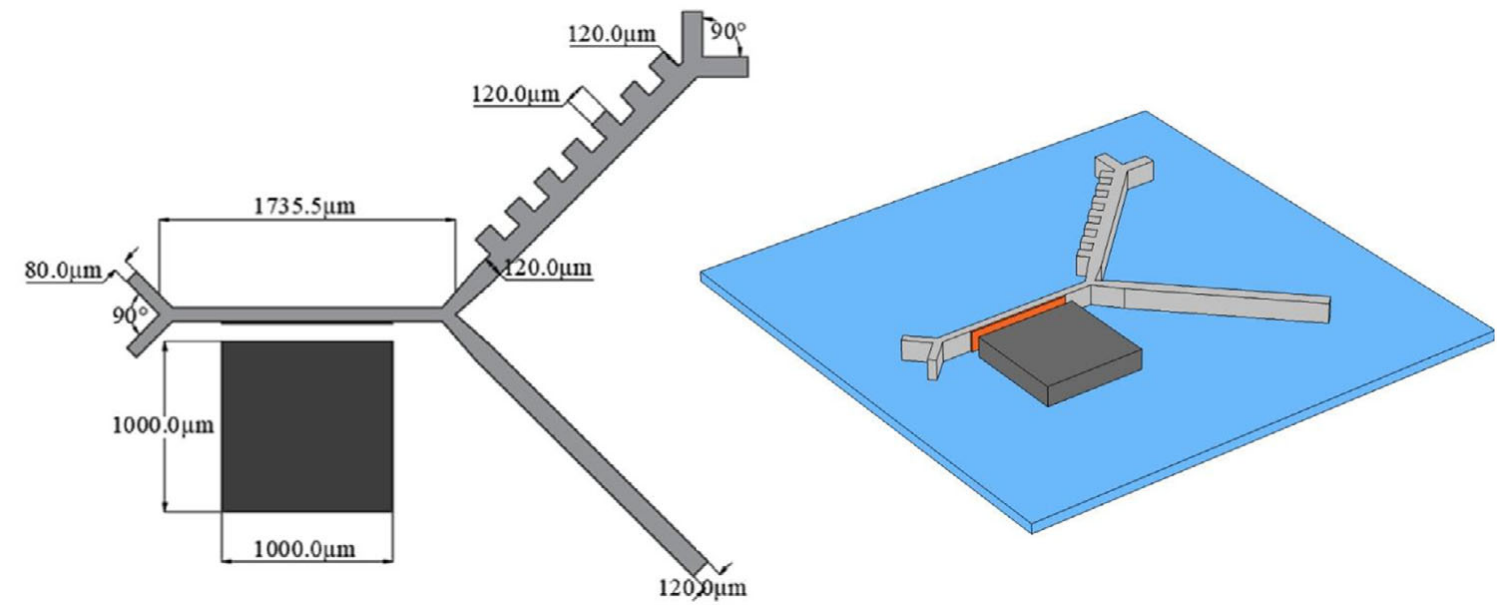
2D and 3D schematics of the device with dimension on 2D

The 3D schematic of the proposed model device is shown in Figure 3. Note that the magnet and electrodes are square shaped with the sides of 1000 µm and 120 µm, respectively, which are appropriate for fabrication. Since the channel depth is larger than its width (*H*/*W* ≈ 5) and Reynolds number is low (< < 1), simulations are done as a 2D modeling. Simulations were done using finite element analysis and they consist of two different studies: steady state and time dependent. The steady‐state study is used for calculating magnetic, electric, and velocity fields within the channel, and the time‐dependent study solves the problem to obtain the trajectories of particles. For the first study, MUMPS is used as the direct solver, while for the latter we used iterative solver GMRES with Jacobi method. Two different triangular grids were generated for solving different fields: An extremely fine mesh for the magnetic and electric fields and a coarser mesh to compute fluid flow. The intention behind using different grids was the necessity to refine the magnetic and electric solutions near singular (sharp) points, while a coarser grid for the fluid flow is sufficient. Grid independency analysis was conducted to ensure that the solutions are independent of the grids. Furthermore, our model was validated by Piacentini et al.’s [6] device, which is shown in Figure S1.

Regarding the fluid flow physics, the device has two inlets defined by average inlet velocities and four outlets with zero pressure boundary condition along with a no‐slip boundary condition for the walls. According to the magnetic physics, the model consists of four different domains; magnet, ferromagnetic track, PDMS mold, and channel. All the domains were defined by the magnetic flux conservation. Constitutive relation of the magnet is set to remanent flux density, while the other domains are set to relative permeability. As the boundary conditions for this physics, magnetic insulation is set to the walls of the PDMS mold. Finally, for the electric currents physics, the walls of the channel are considered as electric insulation, except in the place of the electrodes that are set to an electric potential.

Moreover, particle tracing was conducted using a group of randomly distributed cells at the cell inlet. This group involves four cell lines: 20 PLTs, 20 RBCs, 10 WBCs, and 5 CTCs. Walls and outlets conditions are presumed stick, which means particles will stick to them in the case of collision.

Furthermore, we chose 0.1× PBS as the medium that is widely applicable in DEP separations since it provides sufficient permittivity [6]. PBS is a Newtonian fluid with magnetic susceptibility of water, electrical conductivity of 55 mS/m, and relative permittivity of 80 [6].

**FIGURE 4:**
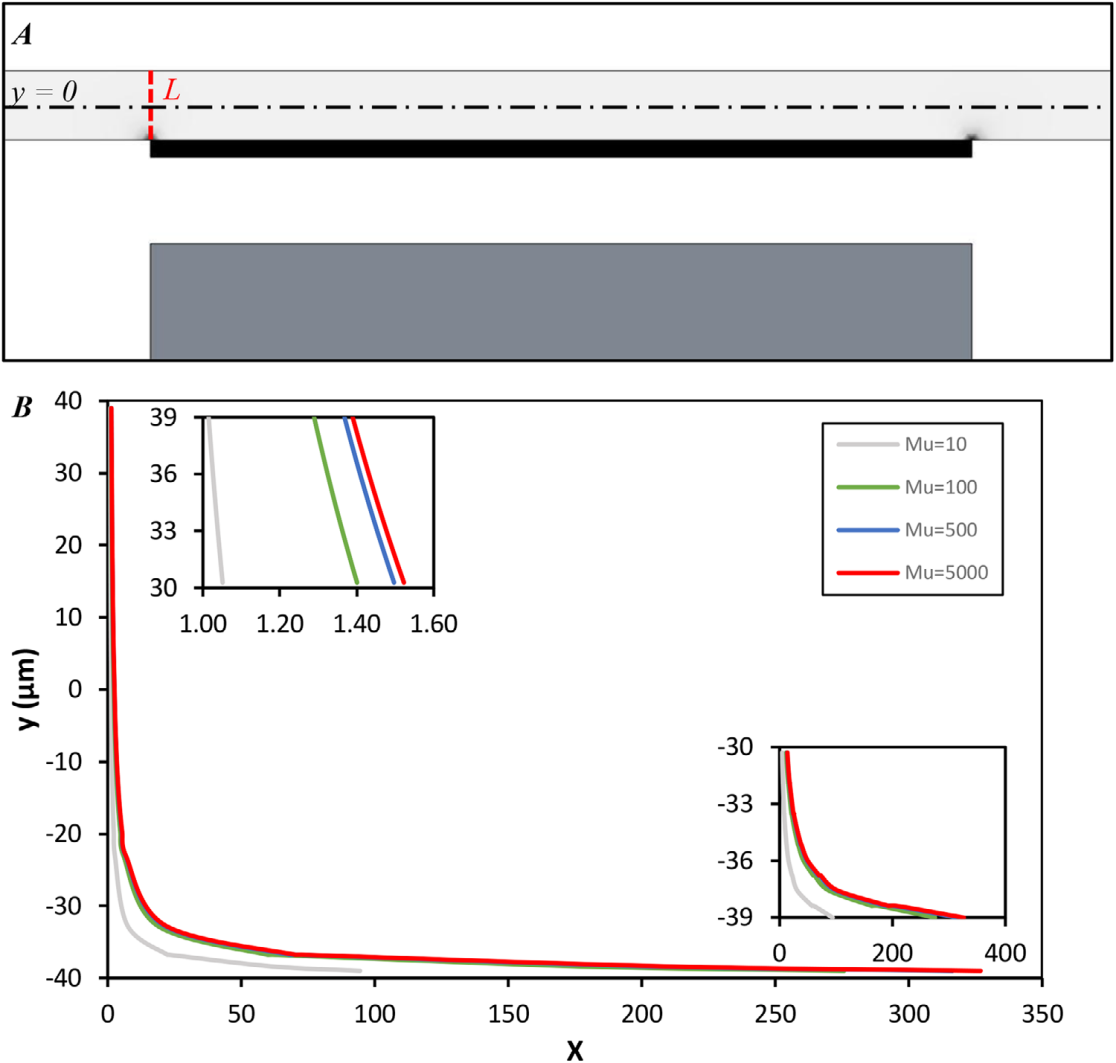
(**A)** H∇B within the channel is demonstrated. Brighter color means lowerH∇B. (**B)** Parameter *X* along line of *L* for different relative permeabilities of 10, 100, 500, and 5000

Equation (3) demonstrates that magnetophoretic force is increased with the increase in the gradient of the magnetic flux density, which is a function of the geometrical spatial frequency, for example, sharpness. Thus, it is anticipated that the effective area of the separation is near the sharp points of the ferromagnetic track, particularly at the start and at the end of the ferromagnetic track. Our model also reports the same results in Figure 4A, where line *L*, shown in this figure, is chosen as the most essential line to analyze the gradient of the magnetic flux density.

To investigate the effect of the ferromagnetic track on the magnetic field within the channel, we solved the model for different relative permeabilities *μ_r_* of ferromagnetic track. Nondimensional parameter *X* is defined as the proportion of H∇B with relative permeability of *μ_r_* to the case with relative permeability *μ_r_* = 1. Note that *μ_r_* = 1 corresponds to the case that the ferromagnetic track is not used, since it is equal to PDMS relative permeability. Figure 4B represents X=H∇B(μ)/H∇B(μ=1) along line *L*. This figure indicates the importance of using ferromagnetic track as it enhances H∇B within the effective area. Also, it can be observed that H∇B does not considerably change with the permeability of 100, 500, and 5000. Therefore, permeability of 500, which is approximately equal to ferrite (nickel–zinc) relative permeability, is assigned to our model.

According to 3, the difference between the cell and the medium susceptibility affects MAP force. Cell radius also plays a crucial role in MAP force. Susceptibility and diameter of different cells are mentioned in Table 1 [13, 14].

**Table 1:** Cells size and magnetic properties

Cell type	Average diameter (μm)	Cell susceptibility (m^3^/kg)	$\chi_{\mathrm{cell}}-\chi_{\mathrm{medium}}\;$ (m^3^/kg)
RBC	6.0	−3.9 × 10^−6^	5.1 × 10^−6^
PLT	2.5	−9.2 × 10^−6^	−0.2 × 10^−6^
HT‐29	11.0	−9.5 × 10^−6^	−0.5 × 10^−6^
WBC	14.0	−9.9 × 10^−6^	−0.9 × 10^−6^

The susceptibilities in this table are evidently less than the water susceptibility (−9 × 10^−6^) except for RBCs. Consequently, PLTs, CTCs, and WBCs experience negative magnetophoretic force that repels them from the ferromagnet track, while positive magnetophoretic force is exerted on RBCs that attracts them to the ferromagnetic track.

Above‐mentioned facts create the potential of separation, but the small difference between susceptibilities of the cells and medium makes the separation more difficult. In order to overcome this difficulty, H∇B must be amplified to increase the MAP force. Figure 4B indicates that a significant MAP force is imposed on the particles that are placed at least 20 μm away from the ferromagnet track. Hence, we used a sheath flow to manipulate the cells toward the ferromagnetic track. For further analysis, we define a velocity ratio parameter VR that shows the proportion of the sheath flow average velocity to the cell flow average velocity. Although VR = 3 is theoretically adequate to locate the cells near the ferromagnet track, it should be noted that the appropriate VR does not completely guarantee the separation. The separation would occur only if an appropriate combination of H∇B, VR, and cell flow average velocity are present.

**FIGURE 5:**
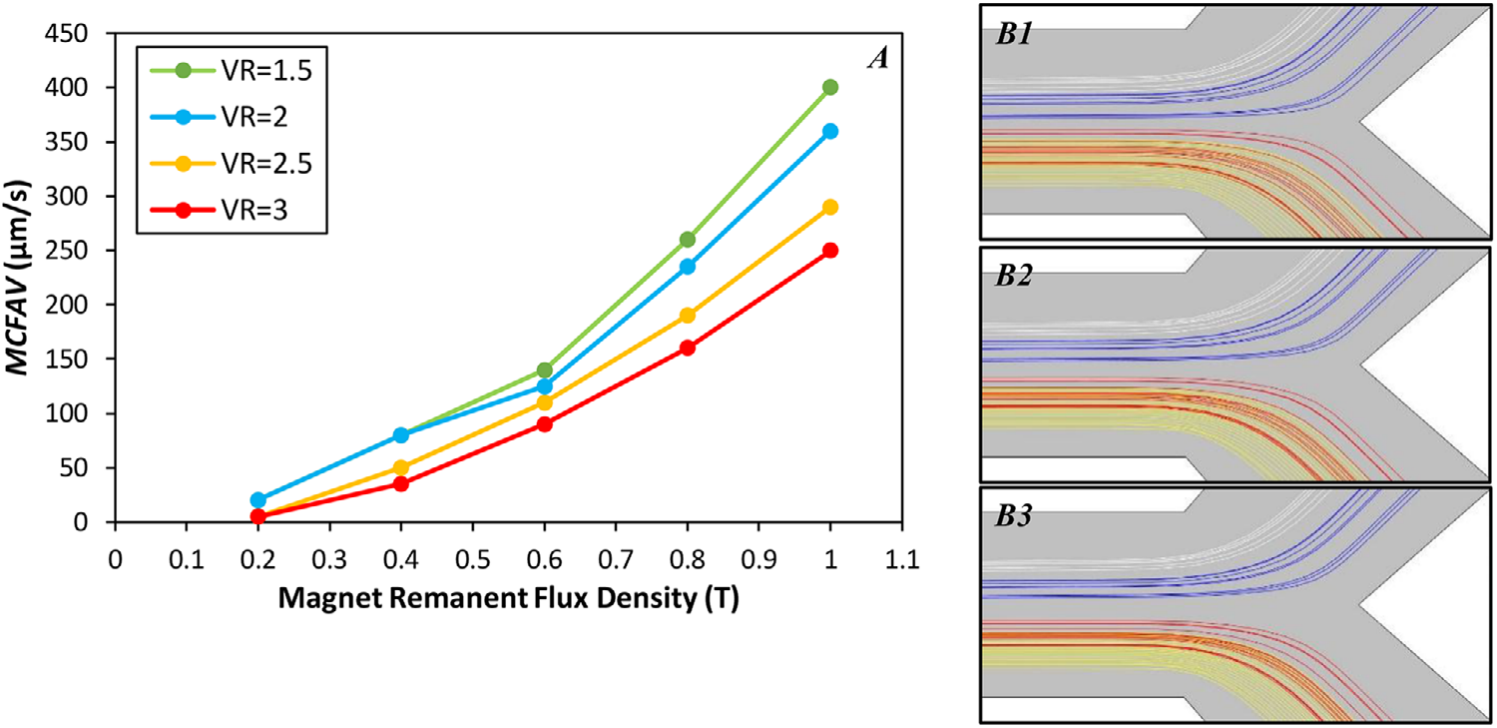
(**A**) Maximum value (to guarantee separation) for cell flow average velocity at five value of remanent flux density of magnet for VR = 1.5, 2, 2.5, and 3. **(B)** Particles trajectories for different VRs with 0.9 MCFAV. White lines are WBCs, blue lines are CTCs (HT‐29), red lines are RBCs, and yellow lines are PLTs trajectories. **B1**: VR = 2, V_Cell‐Flow_ = 200 μm/s. **B2**: VR = 2.5, V_Cell‐Flow_ = 170 μm/s. **B3**: VR = 3, V_Cell‐Flow_ = 140 μm/s

H∇B is also a function of the magnet strength, called remanent flux density (*B_r_*) and the ferromagnet track, which is already set. Therefore, we have to find the optimum values for *B_r_*, VR, and average velocity of the cell flow. First, the separation process was investigated for three different values of VR at 1.5, 2, and 3. For each VR, we plotted the maximum possible cell flow average velocity (MCFAV) for different remanent flux densities in Figure 5A. The trend of these data is compatible with our perception from the Newton's second law, as it shows that the particles with higher velocity require higher MAP force, eventually stronger magnet, to change their directions toward the desired outlet. Also, an increase in VR yields to lower MCFAV. This is simply explainable because of the fact that the flow rate at the place of ferromagnet track corresponds to (1 + VR) × MCFAV. According to this figure, although an increase in VR adversely affects the cell throughput, which is a key parameter in the separation process, it should be noticed that the large VR helps the particle trajectories to be more focused and makes the separation easier. Particle tracing results for different VRs are depicted in Figure 5B, where the cell flow average velocity for each model was approximately adjusted to 0.9 MCFAV in order to ensure the complete separation. This figure shows how bigger VR leads to more focused trajectories.

In this study, we used a 1 mm × 1 mm square magnet. Regarding the results in Figure 5, we set the magnet's remanent flux density to be 0.8 T, which enables the system to work with 260 μm/s (VR* = *1.5), 235 μm/s (VR* = *2.0), 190 μm/s (VR* = *2.5), and 160 μm/s (VR* = *3.0) for the cell flow velocity. However, VR* = *2.5 is the most convenient case since it provides sufficient cell throughput (*V*_Cell‐Flow_ *= *170 μm/s) and proper focused trajectories.

**FIGURE 6:**
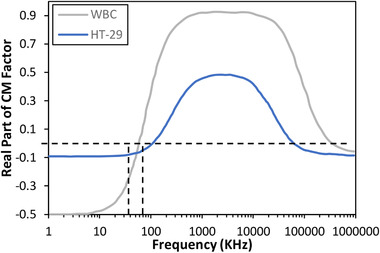
Clausius–Mossoti factor using single shell model

Up to this point, we have successfully separated WBCs and CTCs from RBCs and PLTs. As the next step, WBCs and CTCs should be separated with dielectrophoresis method. There is also a potential for separating RBCs and PLTs from each other, albeit the target cells in the present study are CTCs. Thus, RBCs and PLTs separation in DEP section was not investigated. In DEP section, channel width was increased from 80 to 120 μm that reduced the velocity of the particles. This decreases the electric field within the channel, which is a noticeable point in DEP separation to keep the cells immune. On the other hand, this increase in the width of the channel makes the cell capturing at the outlets easier. The electrode design was inspired from the work of Piacentini et al. [6]. In order to create an electric field, we used electrodes with the same voltage and different signs. In this configuration, pDEP force makes the particles to move toward the electrodes, while nDEP force repels them away from the electrodes. Regarding the previous results from MAP section, WBCs are closer to the electrodes compared to CTCs. Therefore, it is convincing that WBCs experience a pDEP force while nDEP force is imposed on CTCs. Moreover, in the next DEP channel that RBCs and PLTs are going to be separated, PLTs are closer to the electrodes. Hence, pDEP force has to be imposed on PLTs while RBCs experience a nDEP force. As it was mentioned before, the CM factor specifies the sign of DEP force based on the working frequency. CM factor is a function of permittivity and conductivity of the particle and the medium. CM calculation is much more difficult for the cells because they comprise different layers. Single shell model is a conventional approach to obtain the CM factor, although double or triple shell models might yield to more accurate results. CM factor using single shell model for HT‐29s and WBCs versus frequency is shown in Figure 6 [12, 15].

**FIGURE 7:**
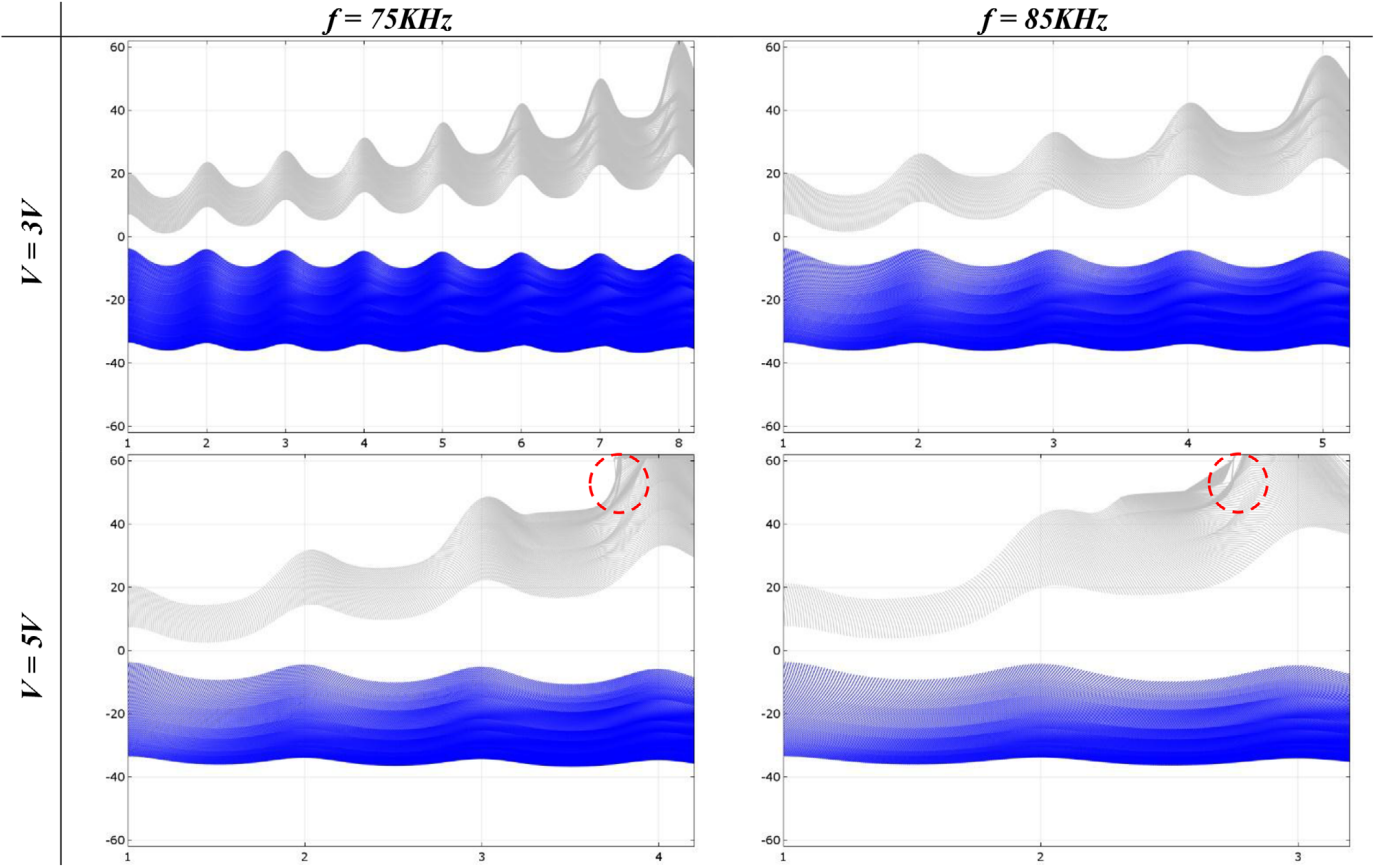
*Y*‐axis is cells width streak in μm, and *X*‐axis is the number of electrodes. Gray lines are WBCs, and CTCs are blue. Red circles show where WBCs come into contact with channel walls

Effective parameters in DEP section are the working voltage, applied frequency, and the number of electrodes. First, WBCs and CTCs have to experience pDEP and nDEP force, respectively. Therefore, CM factor of WBCs should be positive, while CTCs should have a negative *CM* factor. Regarding Figure 6, the applied frequency has to be in the range of 60–100 kHz. There is also another range of frequencies that satisfies the condition, but it starts at the high frequency of 60 MHz considering a feasible fabrication process. CM factor values in the range of 65*–*95 kHz are shown in Table S1. At 60 kHz, DEP force exerted on WBCs would be negligible and the same would happen for CTCs at 100 kHz. Frequencies less than 70 kHz come along with smaller CM factor of WBC, leading to the use of high voltages to compensate for the smaller exerted DEP force. This can also happen for the frequencies higher than 90 kHz for HT‐29. Thus, two frequencies of 75 kHz and 85 kHz are taken into consideration for our further investigation. Second, the voltage could vary in a wide range, but lower working voltages are more desirable, because the cells are vulnerable to high electric field. Hence, we analyze the cell separation with voltages of 3 V and 5 V. Finally, the number of required electrodes to separate WBCs and CTCs depends on the working voltage and the applied frequency. Each set of frequency and voltage leads to a specific number of required electrodes to successfully separate the cells. For each set, the width streak of the cells below the electrodes are shown in Figure 7.

According to Figure 7, CTCs trajectory does not change remarkably under different voltages and frequencies. This is easily explained due to the fact that CTCs are far from electrodes and electric field subsequently becomes unimportant at their place. On the other hand, WBCs are considerably under the influence of voltage and applied frequency, where they promptly approach to the channel wall after three or four electrodes at 5 V. Although *V = *5 V leads to a lower number of required electrodes to separate the cells, we chose 3 V as the working voltage to reduce the electric field and the potential damage to the cells. Note that 85 kHz gives stronger DEP force and might initiate possible contacts to the wall. Hence, the frequency is set to 75 kHz. Using seven electrodes seems sufficient to ensure separation, where WBCs locate at 30 μm with this configuration (*V = *3 V*, f = *75 kHz).

**FIGURE 8:**
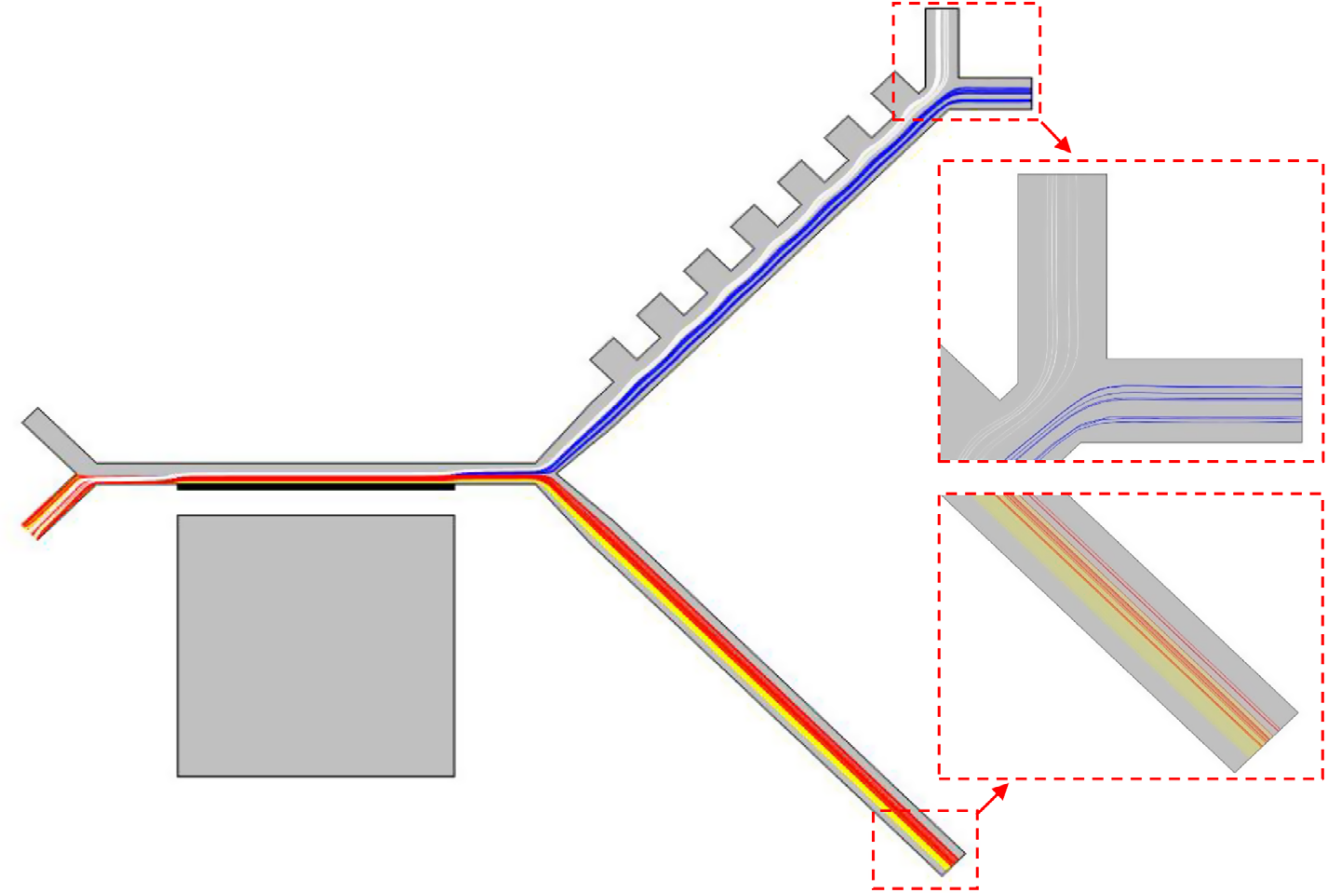
Particle tracing result. Cells are randomly distributed at inlet. Color lines are cells trajectories; yellow, red, blue, and white represent PLTs, RBCs, CTCs, and WBCs trajectories, respectively. Parameters in these simulations are as follows: cell‐flow velocity = 170 μm/s, VR = 2.5, remanent flux density = 0.8 T, V = 3 V, f = 75 kHz

The final trajectories of particles are shown in Figure 8. This integrated simulation indicates complete separation of the cells using our two‐step MAP‐DEP device. RBCs and PLTs were separated using MAP technique from diluted blood sample at the first step, because of their smaller size compared to WBCs and CTCs. At the next stage, WBCs and CTCs were separated from each other with the aid of DEP force. Separation of HT‐29 was investigated in the proposed device, but there is also the potential for the separation of any other types of CTCs that have bigger size compared to RBCs by changing the device voltage and frequency.

## 4 CONCLUDING REMARKS

In our previous studies, different designs of microfluidic devices were implemented for investigating biological phenomena [16, 17, 18, 19, 20, 21, 22]. In this study, a two‐step device including integrated MAP and DEP technique was designed. First, using MAP force, RBCs and PLTs were separated from the blood sample and the remaining cells (WBCs and CTCs) entered the next step, which was the DEP section. PLTs and RBCs also can be separated by DEP technique with the same design. In both sections, cells are separated based on their inherent properties. Label‐free separation of target cells or CTCs is the significant feature of this method and could be easily done either for diagnostic or therapeutic purposes.

We used HT‐29 as CTC in our investigations, but other types of CTCs can be separated using this device as well. The parameters of our studies were based on HT‐29 and the obtained parameters does not guarantee the maximum efficiency and purification for other cell lines.

The use of PBS, instead of the common ferro‐fluids, makes MAP section easy to be utilized. The magnet dimensions and strength have been feasibly selected to make it commercially available.

In DEP section, “liquid electrodes” intrinsically protect the cells from coming into contact with the metal and being exposed to high electric fields, which would definitely harm the cells. Moreover, the use of relatively low voltages reduces the probable damages to the cells. Furthermore, we considered practical and fabrication limitations to propose a feasible device.

## CONFLICT OF INTEREST

The autors have declared no conflict of interest.

## Supporting information

Supporting Information.

## References

[1] Gossett, D. R. , Weaver, W. M. , Mach, A. J. , Claire Hur, S. , et al., Label‐free cell separation and sorting in microfluidic systems. Anal. Bioanal. Chem., 2010, 397, 3249–3267. 20419490 10.1007/s00216-010-3721-9 PMC2911537

[2] Shields, C. W. IV , Reyes, C. D. , López, G. P. , Microfluidic cell sorting: a review of the advances in the separation of cells from debulking to rare cell isolation. Lab Chip. 2015, 15, 1230–1249. 25598308 10.1039/c4lc01246a PMC4331226

[3] Vahey, M. D. , Voldman, J. , An equilibrium method for continuous‐flow cell sorting using dielectrophoresis. Anal. Chem. 2008, 80(9):3135–3143. 18363383 10.1021/ac7020568

[4] Pohl, H. A. , Hawk, I. , Separation of living and dead cells by dielectrophoresis. Science 1966, 152(3722), 647–649. 10.1126/science.152.3722.647-a 17779503

[5] Morgan, H. , Hughes, M. P. , Green, N. G. , Separation of submicron bioparticles by dielectrophoresis. Biophysics 1999, 77, 516–525. 10.1016/S0006-3495(99)76908-0 PMC1300348 10388776

[6] Piacentini, N. , Mernier, G. , Tornay, R. , Renaud, P. , Separation of platelets from other blood cells in continuous‐flow by dielectrophoresis field‐flow‐fractionation. Biomicrofluidics 2011, 5, 034122. 10.1063/1.3640045 PMC3364835 22662047

[7] Valero, T. B. , Demierre, N. , Renaud, P. , A miniaturized continuous dielectrophoretic cell sorter and its applications. Biomicrofluidics. 2010, 4 10.1063/1.3430542 PMC2917879 20697593

[8] Park, S. , Ang, R. R. , Duffy, S. P. , Bazov, J. , et al., Morphological differences between circulating tumor cells from prostate cancer patients and cultured prostate cancer cells. PLoSONE 2014, 9, e85264. 10.1371/journal.pone.0085264 PMC3885705 24416373

[9] Webster, J. G. , Zborowski, M. , Chalmers, J. J. Magnetophoresis: Fundamentals and Applications. In Wiley Encyclopedia of Electrical and Electronics Engineering, J. G. Webster (Ed.). 10.1002/047134608X.W8236.

[10] Wang, X. , Wang, X. ‐ B. , Gascoyne, P. R. , General expressions for dielectrophoretic force and electrorotational torque derived using the Maxwell stress tensor method. J. Electrostat. 1997, 39, 277–295.

[11] Hughes, M. P. , Pethig, R. , Wang, X.‐ B. Dielectrophoretic forces on particles in travelling electric fields. J. Phys. D Appl. Phys. 1996, 29(2), 474–482.

[12] Wu, L. , Yung, L.‐Y. L. , Lim, K. ‐ M. , Dielectrophoretic capture voltage spectrum for measurement of dielectric properties and separation of cancer cells. Biomicrofluidics 2012, 6, 014113. 10.1063/1.3690470 PMC3365349 22662097

[13] Inglis, D. W. , Riehn, R. , Sturm, J. C. , Austin, R. H. , Microfluidic high gradient magnetic cell separation. J. Appl. Phys. 2006, 99 10.1063/1.2165782

[14] Low, W. S. , Kadri, N. A. , Computational analysis of enhanced circulating tumor cell (CTC) separation in a microfluidic system with an integrated dielectrophoretic‐magnetophorectic (DEP‐MAP) technique. MDPI 2016, 4, 14.

[15] Gascoyne, P. R. , Wang, X. ‐ B. , Huang, Y. , Becker, F. F. , Dielectrophoretic separation of cancer cells from blood. IEEE Trans. Ind. Appl. 1997, 33, 670–678 20011619 10.1109/28.585856 PMC2790288

[16] Shamloo, A. , Selahi, A. A. , Madadelahi, M. Designing and modeling a centrifugal microfluidic device to separate target blood cells. J. Micromech. Microeng. 2016, 26(3), 035017.

[17] Shamloo, A. , Kamali, A. , Numerical analysis of a dielectrophoresis field‐flow fractionation device for the separation of multiple cell types. J. Sep. Sci.. 2017, 40, 4067–4075. 28796446 10.1002/jssc.201700325

[18] Shamloo, P. V. , Akbari, A. , Analyzing mixing quality in a curved centrifugal micromixer through numerical simulation. Chem. Eng. Process. 2017, 116, 9–16.

[19] Shamloo, M. B. , Design and simulation of a microfluidic device for acoustic cell separation. Ultrasonics 2018, 84, 234–243. 29175517 10.1016/j.ultras.2017.11.009

[20] Shamloo, A. M , Inertial particle focusing in serpentine channels on a centrifugal platform. Phys. Fluids. 2018, 30, 012002.

[21] Shamloo, M. M. , Abdorahimzadeh, S. , Three‐dimensional numerical simulation of a novel electroosmotic micromixer. Chem. Eng. Process. 2017, 119, 25–33.

[22] Vatankhah, P. , Shamloo, A. , Parametric study on mixing process in an in‐plane spiral micromixer utilizing chaotic advection. Anal. Chim. Acta. 2018, 1022, 96–105. 29729743 10.1016/j.aca.2018.03.039

