# Supplementary material for: Investigation of a two‐step device implementing magnetophoresis and dielectrophoresis for separation of circulating tumor cells from blood cells
Source: Eng Life Sci. 2020 Apr 27;20(7):296–304. doi: 10.1002/elsc.202000001 (PMC7336159; doi:10.1002/elsc.202000001)
Supplement: Supplementary file 1 — Supporting Information. [file ELSC-20-296-s001.docx]

**Supplementary Information**

**Validation**

Figure S1 shows the simulation of *Piacentini et al.* in order to validate our model. In this simulation, cell-flow velocity and sheath-flow velocity were set at 134*µm/s* and 853*µm/s*, respectively. Working voltage and frequency are 5*V* and 100*KHz.* Moreover, the fluid is 0.1X PBS with 55*mS/m* electrical conductivity and 80 relative permittivity.

| 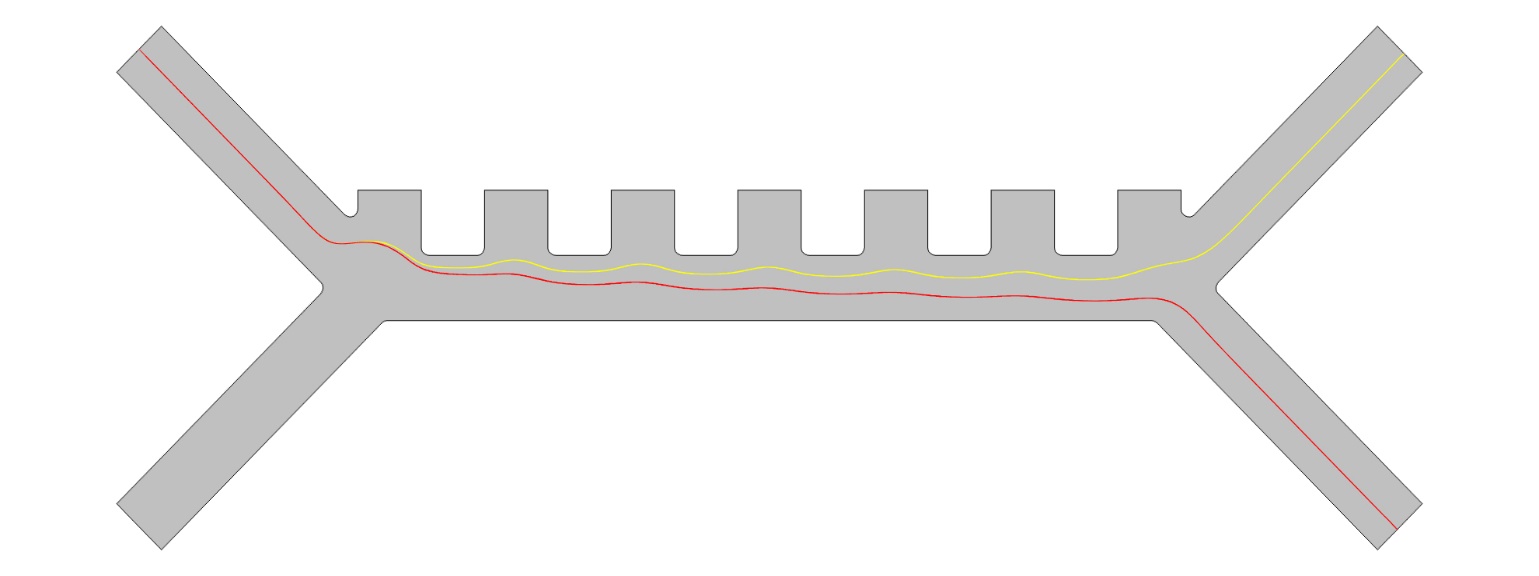 |
| --- |
| **Figure S1.** Validation of the work by Piacentini. Yellow and red lines are PLTs and RBCs, respectively. |

**CM Factor Value**

The Value of CM factor for the range of 65*KHz* to 95*KHz* were obtained from the diagrams and are as the following:

| Table S1. CM values for different frequencies. | | | | | | | |
| --- | --- | --- | --- | --- | --- | --- | --- |
|  | **65*KHz*** | **70*KHz*** | **75*KHz*** | **80*KHz*** | **85*KHz*** | **90*KHz*** | **95*KHz*** |
| *WBC* | 0.065 | 0.120 | 0.155 | 0.235 | 0.265 | 0.300 | 0.330 |
| *HT-29* | -0.055 | -0.050 | -0.041 | -0.032 | -0.022 | -0.017 | -0.008 |
